# Small Extracellular Vesicles Released from Ovarian Cancer Spheroids in Response to Cisplatin Promote the Pro-Tumorigenic Activity of Mesenchymal Stem Cells

**DOI:** 10.3390/ijms20204972

**Published:** 2019-10-09

**Authors:** Nelly Vera, Stephanie Acuña-Gallardo, Felipe Grünenwald, Albano Caceres-Verschae, Ornella Realini, Rodrigo Acuña, Alvaro Lladser, Sebastián E. Illanes, Manuel Varas-Godoy

**Affiliations:** 1 Laboratory of Reproductive Biology, Center for Biomedical Research, Faculty of Medicine, Universidad de Los Andes, Santiago 7620001, Chile; nelly.vera@mayor.cl (N.V.); stephanie.acuna.gallardo@gmail.com (S.A.-G.); felipe.grunenwald@mayor.cl (F.G.); albano.caceres.v@gmail.com (A.C.-V.); or.realini@gmail.com (O.R.); sillanes@uandes.cl (S.E.I.); 2 Centro de Fisiología Celular e Integrativa, Facultad de Medicina, Universidad del Desarrollo, Santiago 7610658, Chile; rodrigo.acuna.bq@gmail.com; 3 Programa de Comunicación Celular en Cáncer, Instituto de Ciencias e Innovación en Medicina (ICIM), Santiago 7610658, Chile; 4 Laboratory of Immunoncology, Fundación Ciencia & Vida, Santiago 7780272, Chile; alladser@cienciavida.org; 5 Department of Obstetrics and Gynaecology, Faculty of Medicine, Universidad de Los Andes, Santiago 7620001, Chile; 6 Cancer Cell Biology Lab, Centro de Biología Celular y Biomedicina (CEBICEM), Facultad de Medicina y Ciencia, Universidad San Sebastián, Lota 2465, Santiago 7510157, Chile

**Keywords:** small extracellular vesicles, cancer stem cells, spheroids, cisplatin, tumor microenvironment, bone marrow mesenchymal stem cells

## Abstract

Despite the different strategies used to treat ovarian cancer, around 70% of women/patients eventually fail to respond to the therapy. Cancer stem cells (CSCs) play a role in the treatment failure due to their chemoresistant properties. This capacity to resist chemotherapy allows CSCs to interact with different components of the tumor microenvironment, such as mesenchymal stem cells (MSCs), and thus contribute to tumorigenic processes. Although the participation of MSCs in tumor progression is well understood, it remains unclear how CSCs induce the pro-tumorigenic activity of MSCs in response to chemotherapy. Small extracellular vesicles, including exosomes, represent one possible way to modulate any type of cell. Therefore, in this study, we evaluate if small extracellular vesicle (sEV) derived from ovarian cancer spheroids (OCS), which are enriched in CSCs, can modify the activity of MSCs to a pro-tumorigenic phenotype. We show that sEV released by OCS in response to cisplatin induce an increase in the migration pattern of bone marrow MSCs (BM-MSCs) and the secretion interleukin-6 (IL-6), interleukin-8 (IL-8), and vascular endothelial growth factor A (VEGFA). Moreover, the factors secreted by BM-MSCs induce angiogenesis in endothelial cells and the migration of low-invasive ovarian cancer cells. These findings suggest that cisplatin could modulate the cargo of sEV released by CSCs, and these exosomes can further induce the pro-tumorigenic activity of MSCs.

## 1. Introduction

According to the World Health Organization, cancer is the leading cause of death worldwide and accounted for 8.8 million deaths in 2015. In the US alone, 1,735,350 new cancer cases and 609,640 cancer deaths are projected to occur in 2018 [1]. Among the different gynecological cancers, ovarian cancer (OVCA) is the most lethal one, and although OVCA accounts for only 2.5% of all malignancies among females, 5% of cancer-related death in women is caused by ovarian cancer [2]. Surgical cytoreduction of the ovarian tumor plus a sequence of chemotherapy has long been considered the best strategy for women with metastatic disease [3]. Although different agents that have been used as first-line chemotherapy (e.g., taxol or platinum) show a significant reduction in the tumor size, around 70% of the women with advanced OVCA relapse within a few years after the treatment and die due to the development of drug resistance [4,5]. Although different therapies such as oncolytic viruses [6] and angiogenesis inhibitors [7], among others have been developed to treat ovarian cancer [8], chemotherapy remains as the most often therapy. A diverse range of molecular and cellular mechanisms has been identified as contributors to the chemoresistance process [9]. Among the cellular components, cancer stem cells (CSCs) are one of the main actors in the development of chemoresistance due to its intrinsic properties [10]. Interestingly, chemotherapy such as cisplatin induces CSC enrichment, favoring a chemoresistance phenotype [11,12]. In the last decade, a group of non-cancer cells present in the tumor microenvironment, also defined as stromal cells, have been proposed to contribute to the acquisition of chemoresistance [13,14], and also to promote tumoral progression in different types of cancer [15,16,17]. Several studies have shown the contribution of bone marrow (BM)-derived cells including mesenchymal stem cells (BM-MSCs) in the formation of the tumor stroma [18,19,20]. In OVCA, MSCs have an important role in the tumor progression, interacting with the tumor cells in response to different stimulus, promoting resistance to therapies [21,22], angiogenesis [23,24], and metastasis [25,26]. It was demonstrated that MSCs associated with ovarian carcinomas could promote tumor growth by increasing the number of CSCs by altered bone morphogenetic protein (BMP) production [27]. Although there is evidence of the contribution of the tumor microenvironment to the maintenance of CSCs population [28], the mechanism of action regarding how CSCs interact and modulate the stromal cells is still unclear. One way to orchestrate different properties of the stroma is possible via a group of secreted membrane structures called extracellular vesicles (EVs). EVs can be classified in two main groups, microvesicles (vesicles derived from the plasma membrane with a range of size between 50–1000 nm in diameter) and exosomes (vesicles derived from the endosomal membrane with a range of size between 30–100 nm in diameter) [29]. Due to the difficulty of differentiating exosomes and microvesicles of the same size, the International Society for Extracellular Vesicles (ISEV) called vesicles smaller than 200 nm in diameter small EVs (sEV) [30]. Small EVs, including exosomes, can transfer their contents (proteins, long RNAs, microRNAs, lipids, etc.) from a cancer cell to a target cells and thus change its phenotype regulating diverse biological processes [29,31]. Additionally, due to the natural delivery and targeting properties of the EVs, these can be used as a drug delivery system of different compounds, including chemotherapy [32,33,34]. Depending on the cell that releases the EVs, and its specific contents, EVs can be used as a cancer liquid biopsy [35,36,37]. Although in cancer, the role of tumor-derived extracellular vesicles as critical mediators of cellular communication and tumor progression is well reported in the literature [38,39,40,41], the specific contribution of sEV derived from CSCs in the modulation of the tumor microenvironment, and the effect of chemotherapy on the activity of the CSCs-derived sEV, remain poorly understood.

In this study, we evaluated the effect of sEV derived from ovarian cancer spheroids (OCS), which are enriched in CSCs, in response or not to cisplatin, on the induction of BM-MSCs with pro-tumorigenic activity. We show that sEV derived from OCS in response to cisplatin (sEV-OCS-Cis) induce an increase in the migration of BM-MSCs accompanied by an increase in the gene expression of metalloproteinases. Moreover, the BM-MSCs in response to the sEV-OCS-Cis have the capability to induce angiogenesis in endothelial cells and migration in low-invasive ovarian cancer cells. These findings suggest that cisplatin modulates the small extracellular vesicles release by CSCs, and these sEV can induce the pro-tumorigenic activity of MSCs.

## 2. Results

### 2.1. Characterization of Exosomes Released by Ovarian Cancer Spheroids in Response to Cisplatin

**Figure 1:**
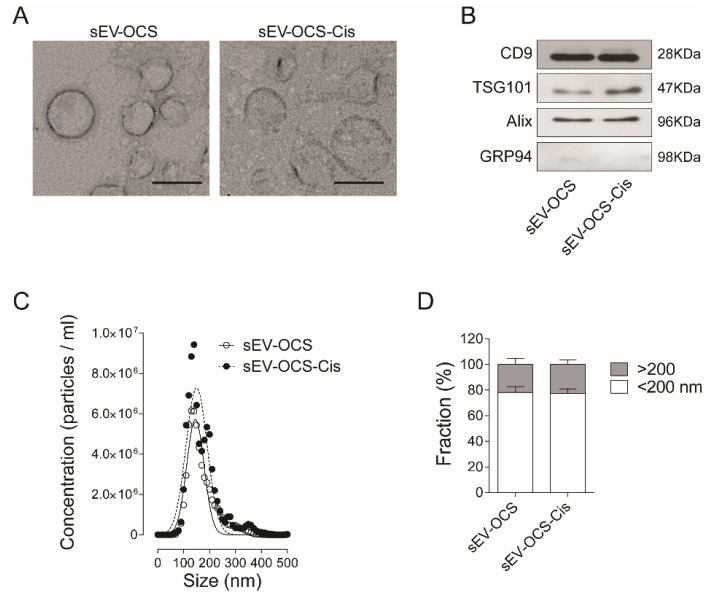
Characterization of small extracellular vesicles dervied from ovarian cancer spheroids (sEV-OCS) and sEV derived from OCS in response to cisplati (sEV-OCS-Cis). (**A**) Transmission electron microscopy of sEV-OCS-Cis (right panel) and sEV-OCS (left panel). Bar 100 nm. (**B**) Western blot of positive markers of exosomes (CD9, TSG101, Alix) in sEV-OCS and sEV-OCS-Cis. As a control of exosome purity, a marker of endoplasmic reticulum (GRP94) was used. (**C**,**D**) Distribution size of sEV-OCS (white circles) and sEV-OCS-Cis (black circles) measured by nanoparticles tracking analysis. Data represent the mean of three independent experiments.

Small EVs derived from OCS were isolated by ultracentrifugation from cell-cultured media (CM) of HeyA8 cells cultured under spheres-forming conditions treated with or without cisplatin. The typical cup shape of EVs was observed in both conditions (Figure 1A), and the presence of exosomal markers CD9, Alix, TSG101, and the absence of the endoplasmatic reticulum marker GRP94 were confirmed by Western blot (Figure 1B). When we compared the distribution size of the sEV by nanoparticle tracking analysis, we did not observe any difference in the sEV release by OCS in response to cisplatin in comparison with the non-treated cells (Figure 1C). When we analyzed the percentage of EVs enriched in our preparation, we observed around 80% of EVs smaller than 200 nm in both sEV-OCS and sEV-OCS-Cis (Figure 1D).

### 2.2. Small EVs Released from Ovarian Cancer Spheroids in Response to Cisplatin Induce Migration and Increase in the Metalloproteinases Expression of Bone Marrow Mesenchymal Stem Cells

**Figure 2:**
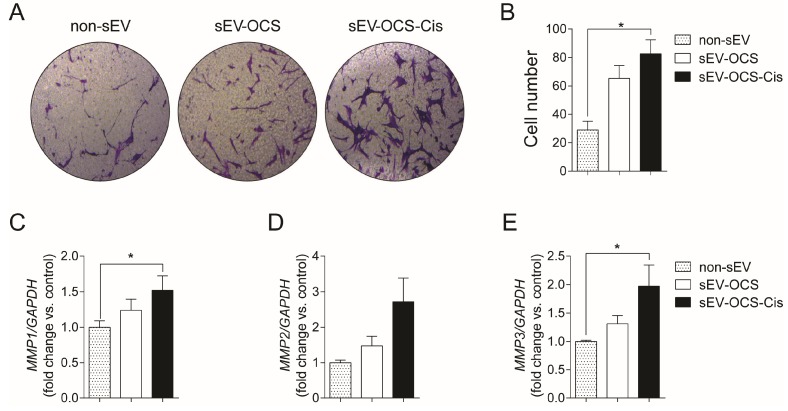
Migration of bone marrow mesenchymal stem cells (BM-MSCs) after stimulation with sEV-OCS or sEV-OCS-Cis. (**A**) Representative images of migration assay performed by transwell of BM-MSCs stimulated with sEV-OCS or sEV-OCS-Cis. (**B**) Quantification of BM-MSCs migration stimulated with sEV-OCS or sEV-OCS-Cis. Migrated cell numbers were determined by counting the number of cells contained in the photos of five different fields at original magnification 10x. (**C**–**E**) Relative fold change of (**C**) metalloproteinases 1 (MMP1), (**D**) MMP2, and (**E**) MMP3 expression of BM-MSCs stimulated with sEV-OCS or sEV-OCS-Cis measured by RT-qPCR. BM-MSCs without sEV stimulation (non-sEV) were used as a control in all the experiments. Results are mean ± SEM (standard error of the mean) of *n* = 4. Statistical analysis was performed using the Kruskal–Wallis test followed by Mann–Whitney test. * *p* < 0.05.

Since MSCs are important for the formation of the tumor stroma, we evaluate the effect of sEVs released from OCS on the migration of BM-MSCs. We cultured BM-MSCs with sEV-OCS treated with or without cisplatin, in a ratio of 10,000 exosomes per cell, for 16 hours, and then seeded them in a Boyden chamber to obtain the migration pattern of the BM-MSCs. sEV-OCS-Cis induce a significance increase in the migration of BM-MSCs in comparison with BM-MSCs cultured without sEV (Figure 2A,B). This effect was not observed in the BM-MSCs cultured with the sEV-OCS (Figure 2A,B). The higher migration in the BM-MSCs cultured with the sEV derived from OCS was accompanied by an increase in the expression of the metalloproteinases (MMP) 1, 2, and 3 (Figure 2C–E). These data suggest that cisplatin could change the cargo of sEV released from ovarian cancer spheroids and induce a change in the migration pattern of BM-MSCs.

### 2.3. BM-MSCs Stimulated with Small EVs Derived from Ovarian Cancer Spheroids in Response to Cisplatin Secrete Higher Levels of IL-6, IL-8, and VEGFA

**Figure 3:**
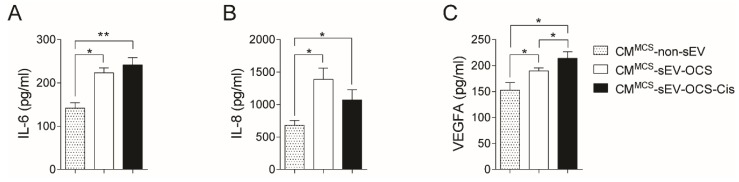
Secretion of interleukin-6 (IL-6), interleukin-8 (IL-8), and vascular endothelial growth factor A (VEGFA) from BM-MSCs after stimulation with sEV-OCS or sEV-OCS-Cis. (**A**–**C**) Secretion levels of (**A**) IL-6, (**B**) IL-8, and (**C**) VEGFA of BM-MSCs stimulated with sEV-OCS or sEV-OCS-Cis measured by multiplex fluorescent bead-based immunoassay analysis. BM-MSCs cell-cultured media (CM) was harvested 24 hours post-stimulation with sEV-OCS (CM^MCS^-sEV-OCS) or sEV-OCS-Cis (CM^MCS^-sEV-OCS-Cis). CM of BM-MSCs without sEV stimulation (CM^MCS^-non-sEV) was used as control. Results are mean ± SEM of *n* = 3. Statistical analysis was performed using the Kruskal–Wallis test followed by the Mann–Whitney test. * *p* < 0.05, ** *p* < 0.01.

Since the secretion of several factors from MSCs is a key component of cancer progression [42,43], we evaluated in the conditioned media of BM-MSCs stimulated with small EVs derived from OCS the levels of three factors (interluekin-6 (IL-6), interleukin-8 (IL-8), and vascular endothelial growth factor A (VEGFA)) involved in pro-tumorigenic processes. Both BM-MSCs stimulated with sEV derived from OCS treated or not with cisplatin increased significantly the secretion of IL-6 and IL-8 in comparison with non-stimulated BM-MSCs (Figure 3A,B). In the case of VEGFA, only the CM of BM-MSCs stimulated with sEV-OCS-Cis showed higher levels of VEGFA in comparison with the BM-MSCs without stimulation (Figure 3C).

### 2.4. BM-MSCs Stimulated with Small EVs Derived from Ovarian Cancer Spheroids in Response to Cisplatin Induce Angiogenesis

**Figure 4:**
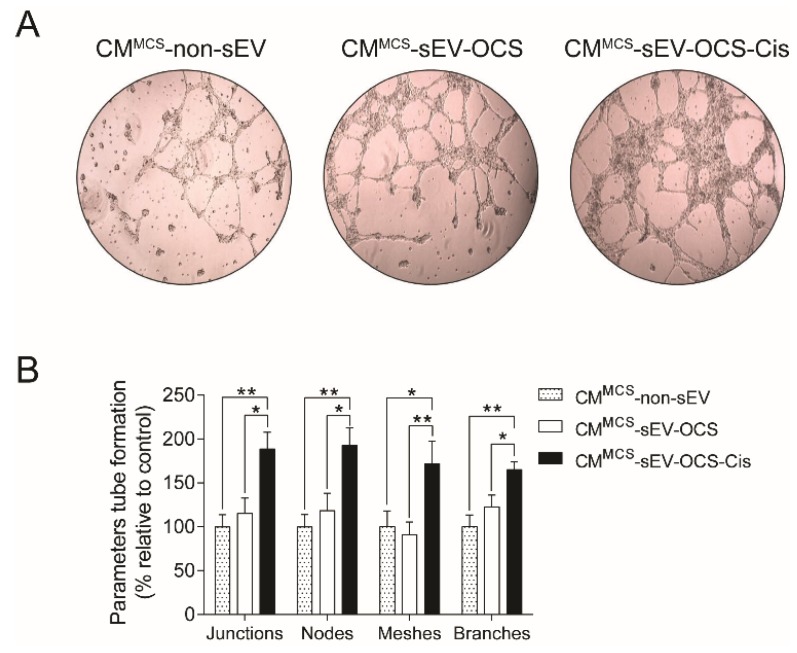
Pro-angiogenic properties of BM-MSCs after stimulation with sEV-OCS or sEV-OCS-Cis. (**A**) Representative images of tube formation assay performed with human umbilical vein endothelial cells (HUVEC) cells grown in Matrigel stimulated with CM^MCS^-sEV-OCS or CM^MCS^-sEV-OCS-Cis. (**B**) Quantification of tube formation parameters (junctions, nodes, meshes, and branches) of HUVEC cells stimulated with CM^MCS^-sEV-OCS or CM^MCS^-sEV-OCS-Cis. Tube formation was examined by phase contrast. Images were captured using an Olympus U-RFL-T camera. The tube formation parameters were analyzed using the ImageJ Angiogenesis Analyzer software. CM of BM-MSCs without sEV stimulation (CM^MCS^-non-sEV) was used as control. Results are mean ± SEM of *n* = 6. Statistical analysis was performed using the Kruskal–Wallis test followed by the Mann–Whitney test. * *p* < 0.05, ** *p* < 0.01.

To demonstrate if the secreted factors released by the BM-MSCs stimulated with small EVs derived from OCS promote pro-tumorigenic properties, we assessed the capability of BM-MSCs to induce angiogenesis. Human umbilical vein endothelial cells (HUVEC) cells were cultured in Matrigel with CM derived from BM-MSCs stimulated with sEV, and then the tube formation was analyzed. We observed that only BM-MSCs stimulated with sEV-OCS-Cis increase significantly the angiogenic properties of BM-MSCs in comparison with the BM-MSCs stimulated with sEV derived from ovarian cancer spheroids without the treatment of cisplatin or the BM-MSCs without stimulation with sEV (Figure 4A,B).

### 2.5. BM-MSCs Stimulated with Small EVs Derived from Ovarian Cancer Spheroids in Response to Cisplatin Induce Migration of Low-Invasive Ovarian Cancer Cells

**Figure 5:**
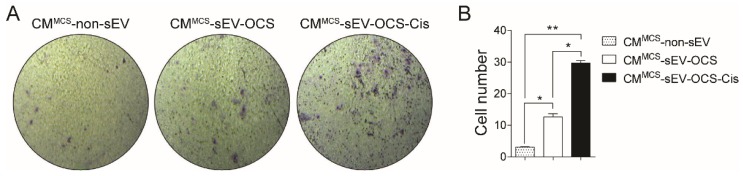
Pro-migratory tumor properties of BM-MSCs after stimulation with sEV-OCS or sEV-OCS-Cis evaluated in low-invasive ovarian cancer cells (Ovcar3). (**A**) Representative images of migration assay performed by transwell of Ovcar3 cells stimulated with CM^MCS^-sEV-OCS or CM^MCS^-sEV-OCS-Cis. (**B**) Quantification of Ovcar3 cells migration stimulated with CM^MCS^-sEV-OCS or CM^MCS^-sEV-OCS-Cis. Migrated cell numbers were determined by counting the number of cells contained in the photos of five different fields at original magnification 10x. CM of BM-MSCs without sEV stimulation (CM^MCS^-non-sEV) was used as control. Results are mean ± SEM of *n* = 4. Statistical analysis was performed using the Kruskal–Wallis test followed by the Mann–Whitney test. * *p* < 0.05, ** *p* < 0.01.

Finally, we also asked if small EVs released from OCS can induce another type of pro-tumorigenic activity in BM-MSCs. For that, we performed a transwell migration assay in low-invasive ovarian cancer cells (Ovcar3) cultured with the CM derived from BM-MSCs stimulated with sEV. Similar to the angiogenic properties, only the BM-MSCs stimulated with sEV-OCS-Cis induced a significant increase in the migration of Ovcar3 cells in comparison with the other groups (Figure 5A,B).

## 3. Discussion

Here, we showed that small extracellular vesicles released from ovarian cancer spheroids enriched in CSCs in response to the chemotherapeutic agent cisplatin promote a more pro-tumorigenic phenotype of BM-MSCs. The evidence in the literature supports the importance of CSCs in the tumor progression of different types of cancer due to its role in drug resistance [10] and metastasis [44]. These characteristics are supported by the interaction of the CSCs with the tumor microenvironment (TME), which can contribute to the CSCs maintenance.

In ovarian cancer, MSCs contribute to the tumor progression increasing the number of CSCs regulating the bone morphogenetic protein (BMP) signaling [27]. These interactions can recruit the MSCs into the tumor, educate them to acquire a pro-tumorigenic phenotype, and promote tumor progression [45,46]. The crosstalk between ovarian CSCs and the different components of the TME, including MSCs, is mediated by several types of molecules such as cytokines, growth factors, and lipids [28]. In the last decade, small extracellular vesicles, including exosomes, have taken relevance in cancer as cell-to-cell mediators due to the ability of tumor-derived extracellular vesicles to carry and deliver a malignant cargo (proteins, lipids, long non-coding RNAs, and microRNAs) which can reprogram recipient cells [39]. In line with the concept that EVs’ cargo depends on the tumor cell origin and state, cisplatin could change the type of EVs secreted by ovarian CSCs, and therefore modulate the recipient cell differently. When we characterized the extracellular vesicles derived from ovarian cancer spheroids according to the recommendations of the International Society for Extracellular Vesicles (ISEV) [30], we confirm that our preparation was enriched in small EVs, showing around 80% EVs smaller than 200 nm. In terms of physical and biochemical characteristics, we did not observe differences in the morphology, typical protein markers, and size distribution of the small EVs released from ovarian cancer spheroids (sEV-OCS) and ovarian cancer spheroids treated with cisplatin (sEV-OCS-Cis) (Figure 1). Although it has been reported that chemotherapy induces an increase in the secretion of EVs from tumor cells [47,48], we could not confirm this evidence using cisplatin in the OCS, because we could not calculate the number of cells derived from the spheres in the moment of the sEV isolation.

In terms of extracellular vesicles activity, OCS released small EVs in response to cisplatin, which in turn were able to induce the pro-tumorigenic phenotype of BM-MSCs. Lindoso and collaborators showed that EVs derived from renal CSCs after two weeks of stimulation induced a pro-tumorigenic phenotype in MSCs, promoting a higher migration rate of the MSCs, and a higher capability of the MSCs to induce the angiogenesis of endothelial cells and migration of renal tumor cells [49]. In our work, we observed the same effect mentioned in the previous work using small EVs derived from ovarian cancer spheroids in response to cisplatin (sEV-OCS-Cis). We also observed an increase in the BM-MSCs migration rate, which was accompanied by an increase in the expression of MMP1 and MMP3 after stimulation with the sEV (Figure 2). Finally, these BM-MSCs also present higher pro-tumorigenic activity in comparison with the non-stimulated cells (Figure 4 and Figure 5). Although our results did not show an increase in the pro-tumorigenic activity of BM-MSCs after the stimulation with small EVs derived from OCS (sEV-OCS), the stimulation was only for 24 hours. A longer stimulation with our sEV-OCS could probably increase the pro-tumorigenic activity of the MSCs in our model. Another reason explaining why we did not observe effects using sEV-OCS could be because our spheroids are only enriched, and are not a pure population of CSCs; therefore, the isolation of sEV derived from a pure population of CSCs could increase the effects of the sEV. Interestingly, both sEV-OCS and sEV-OCS-Cis induced an increase in the secretion of IL-6, IL-8, and VEGFA from BM-MSCs (Figure 3). These factors secreted by MSCs are key inductors of pro-tumorigenic processes such as angiogenesis [50,51], CSCs self-renewal [52], and metastasis [53,54]. Therefore, the pro-tumorigenic activity observed only in the conditioned media of MSCs stimulated with sEV-OCS-Cis could be attributed to other factors secreted by the MSCs. Interestingly, in the case of IL-8 secretion, we observed a higher secretion of this cytokine from the BM-MSCs stimulated with sEV-OCS (Figure 3B). In some types of cancer, the signaling mediated by IL-8 and its receptor CXCR1 (C-X-C chemokine receptor type 1) is associated with CSCs properties [55,56,57]; therefore, the induction of IL-8 secretion by MSCs mediated by sEV-OCS could be support the self-renewal and maintenance of the CSCs niche in ovarian cancer.

Unpublished data of our group using ovarian cancer chemoresistance cells (A2780cis) also show that sEV derived from these cells (sEV-A2780cis-Cis) induce the migration of BM-MSCs in comparison with sEV derived from non-treated cells (sEV-A2780cis) [58]. This result is supported by the evidence from the literature demonstrating that chemotherapy induces the secretion of small EVs with pro-tumorigenic activity [59,60,61].

Some reports have described the direct effect of CSCs-exosomes transferring their properties to cancer cells and promoting tumor progression. In colorectal cancer (CRC), CSC-derived exosomes promote the increase of CSCs via the transfer of the microRNA miR-146a-5p, which downregulates the expression of NUMB [62], a tumor suppressor gene regulating the Notch pathway [63]. In renal cancer, it has been shown that microvesicles derived from CSCs stimulate angiogenesis and the formation of the lung pre-metastatic niche [64]. Moreover, renal CSCs-exosomes induced the epithelial mesenchymal transition (EMT) of clear cell renal cell carcinoma through the transfer of the microRNA miR-19b-3p, which promotes lung metastasis [65]. These data support that CSCs-exosomes can reflex the stem cell state of the tumor cell and transfer its activity to non-CSCs to modulate its phenotype to promote tumor progression.

Taken together, this study shows that chemotherapy such as cisplatin can modulate the release of small EVs from ovarian cancer cells with CSCs characteristics and promote a pro-tumorigenic activity of BM-MSCs. Its effects could be mediated by changes in the cargo of small EVs in response to the activation of several pathways in the CSCs by cisplatin. Futures studies are needed to understand which pathways can modulate the cargo of extracellular vesicles in CSCs, and on the other hand, which are the components of this cargo able to transfer the pro-tumorigenic activity to a recipient cell.

## 4. Materials and Methods

### 4.1. Cell Culture

Ovarian cancer HeyA8 cells (donated by Dr. Gareth Owen, Pontificia Universidad Católica de Chile) and Ovcar3 cells (ATCC, American Type Culture Collection, Manassas, VA, USA) were cultured in RPMI-1640 medium containing 10% fetal bovine serum (FBS) and 1% pen-strep (all Gibco, ThermoFisher Scientific, Waltham, MA, USA). Bone marrow mesenchymal stem cells (BM-MSCs, Lonza, Cleveland, TN, USA) were cultured in DMEM (Gibco, ThermoFisher Scientific, Waltham, MA, USA) containing 10% FBS and 1% pen-strep. All cells were grown at 37 °C in a humidified atmosphere incubator of 95% air and 5% CO_2_. Human umbilical vein endothelial cells (HUVEC, donated by Dr. Maroun Khoury, Cells for Cells).

### 4.2. Ovarian Cancer Stem Cells Enrichment

To obtain a cell culture enriched in CSCs, we cultured ovarian cancer cells in spheres conditions under hypoxia [66,67,68]. Briefly, single HeyA8 cells were cultured in ultra-low attachment Petri dishes (Corning Incorporated, Corning, NY, USA) at a density of 10^5^ cells/mL in the stem cell medium (SCM): DMEM/F12 (1:1), 20 ng/mL epidermal growth factor (EGF; Invitrogen), 10 ng/mL basic fibroblast growth factor (bFGF; Sigma-Aldrich, San Luis, MO, USA), 5 ug/mL insulin (Sigma-Aldrich, San Luis, MO, USA), 1% antibiotic-antimycotic solution, and under hypoxic conditions (1% O_2_) for 7 days in the hypoxic chambers C474 equipped with Pro-Ox 110 oxygen controlling regulator (BioSpherix, Parish, NY, USA).

### 4.3. Chemotherapeutic Treatment

After seven days of sphere formation, the medium was replaced by fresh SCM without (control) or with (treated) cisplatin (5 µM; Sigma-Aldrich, San Luis, MO, USA). After 72 hours, the medium was recovered for sEV isolation.

### 4.4. Small Extracellular Vesicles Isolation

Small extracellular vesicles (sEV) derived from ovarian cancer spheroids non-treated (sEV-OCS) or treated with cisplatin (sEV-OCS-Cis) were isolated from cell-cultured media (CM) by differential ultracentrifugation, as previously described [69]. CM was centrifuged at: (i) 300× *g* for 10 min 4 °C, (ii) 2000× *g* for 20 min 4 °C, (iii) 10,000× *g* for 40 min 4 °C, and (iv) 100,000× *g* for 90 min at 4 °C. After each step, the pellet was discarded, and the supernatant was used for the following step. The resulting pellet after the fourth step was resuspended in PBS, filtered with a 0.2-µm filter, and washed at 100,000× *g* for 90 min 4 °C to remove any contaminating proteins. The pellet was then resuspended in PBS.

### 4.5. Transmission Electron Microscopy

The sEV-OCSLCs and sEV-OCSLCs-Cis isolated by ultracentrifugation were examined by transmission electron microscopy (TEM) to evaluate the morphology of the sEV. The samples were fixed with paraformaldehyde (PFA), deposited in a TEM Formvar carbon grid, and contrasted with uranyl acetate [70]. The sEV were visualized in a Philips Tecnai 12 electron microscope (Pontificia Universidad Católica de Chile).

### 4.6. Western Blot

Exosome markers were identified by Western blot in sEV-OCS and sEV-OCS-Cis. In brief, 10 μg of sEV proteins were separated on a 12% polyacrylamide gel, and proteins were transferred to polyvinylidene difluoride membrane (PVDF; ThermoFisher Scientific, Waltham, MA, USA) in transfer buffer for 1 hour at 100 V. The membrane was washed in washing buffer (PBS TWEEN 0.1%) and blocked with 5% skimmed milk in PBS TWEEN (0.1%) for one hour at room temperature under agitation. The antibodies used to identify exosome-specific proteins were: anti-CD9 (1:1000, Abcam ab65230), anti-Alix (1:1000, Sta. Cruz sc-53540), anti-TSG101 (1:1000, Sta. Cruz sc-7964), and GRP94 (1:1000, Abcam ab90458) as a negative control. The membrane was incubated in primary antibody diluted in 5% skim milk in PBS TWEEN (0.1%) at 4 °C overnight under agitation. After overnight incubation, the membrane was washed and exposed to the appropriated secondary antibody. The membrane was washed three times for 10 min, and the bound antibodies were detected using horseradish peroxidase linked to anti-rabbit or anti-mouse conjugates as appropriate (KPL) and visualized using an Enhanced chemiluminescence (ECL) detecting system (ThermoFisher Scientific, Waltham, MA, USA).

### 4.7. Nanoparticles Tracking Analysis

The size distribution and concentration of sEV-OCS and sEV-OCS-Cis were analyzed using NanoSight NS300 equipment (Malvern Instruments, Malvern, United Kingdom). The analysis was performed using the 532-nm laser and 565-nm long pass filter, with a camera level at 9 and detection threshold of 3. Small EVs were diluted in PBS before the analysis.

### 4.8. Mesenchymal Stem Cells Migration

BM-MSCs were cultured with sEV-OCS or sEV-OCS-Cis in a ratio of 10,000 sEV per cell. After 16 hours of stimulation, the BM-MSCs were tripsinized and cultured in a transwell migration assay (Corning Incorporated, Corning, NY, USA). Twenty-four hours after cell seeding, the BM-MSCs of the lower surface of the membrane (migrated cells) were fixed using methanol and stained with crystal violet (0.2%). Images were taken in a light microscope with 10 × magnification, and the numbers of migrated cells were counted in five different fields per image.

### 4.9. Reverse Transcription-Quantitative Polymerase Chain Reaction

Total RNA from BM-MSCs cultured with sEV-OCS or sEV-OCS-Cis was isolated with Trizol Reagent (Life Technologies, Carlsbad, CA, USA) 24 hours after stimulation. The RNA was quantified by Nanodrop (ThermoFisher Scientific, Waltham, MA, USA), and the complementary DNA (cDNA) was generated using the SuperScript™ II Reverse Transcriptase (Invitrogen, Carlsbad, CA, USA). The reverse transcription (RT) was performed in 12 µL of reaction: 1 µL of deoxynucleotides (dNTP) (10 mM), 1 µL of random primers (100 ng/µL), 1 µg of total RNA treated with DNAse, and nuclease-free water. The mix was incubated to 65 °C for 5 min and after the incubation was added to the mix, 4 µL of 5 × first-strand buffer, 2 µL of Dithiothreitol (DTT) (0,1 M), and 1 µL of RNAseOUT (30U µL). The mix was incubated to 25 °C for 2 min, and 1 µL of SuperScript™ II Reverse Transcriptase (200U) was added. The complete reaction was incubated to 25 °C for 10 min, 42 °C for 50 min, and inactivated to 70 °C for 15 min. The quantitative real-time PCR (qPCR) was performed using the Brilliant II QPCR Master Mix (Agilent Technologies, Santa Clara, CA, USA) mixing 2.5 µL of diluted cDNA, 200 nmoles of each primer, 5 µL of Brilliant II QPCR Master Mix, and nuclease-free water in a final volume of 10 µL. The reaction was incubated 10 min to 95 °C, 40 cycles of 20 sec to 95 °C, 20 sec to 60–62 °C, 20 sec to 72 °C, and finally 10 sec to 95 °C, 5 sec to 25 °C, 1 sec to 55 °C, and 1 sec to 95 °C in the Stratagene Mx3000P system (Agilent Technologies, Santa Clara, CA, USA). The primers used to analyze the expression of the migration-related genes were: MMP1: Forward 5’-GGGAGATCATCGGGACAACTC-3’, Reverse 5’-GGGCCTGGTTGAAAAGCAT-3’, MMP2: Forward 5’-TGTGACGCCACGTGACAAG-3’, Reverse 5’-CCAGTATTCATTCCCTGCAAAGA-3’, and MMP3: Forward 5’-CTGGACTCCGACACTCTGGA-3’, Reverse 5’-CAGGAAAGGTTCTGAAGTGACC-3’. Glyceraldehyde 3-phosphate dehydrogenase (GAPDH) was used as a housekeeping gene: Forward 5’-GGAAGATGGTGATGGGATTTC-3’, Reverse 5’-GAAGGTGAAGGTCGGAGTCAA-3’. All the primers were synthesized by IDT DNA technologies (Coralville, IA, USA). Transcript levels were quantified using the comparative CT method.

### 4.10. Luminex Assay

Secreted factors were measured using a commercial multiplex fluorescent bead-based immunoassay (R&D Systems, Minneapolis, MN, USA) following the manufacturer’s instructions. The concentrations of interleukin-6 (IL-6), interleukin-8 (IL-8), and vascular endothelial growth factor A (VEGFA) were determined in CM of BM-MSCs stimulated with sEV-OCS or sEV-OCS-Cis for 24 hours using a MAGPIX System (ThermoFisher Scientific, Waltham, MA, USA) as was described previously [71].

### 4.11. Tube Formation Assay

Human umbilical vein endothelial cells (HUVECs) were cultured in plates coated with Matrigel (Corning Incorporated, Corning, NY, USA) with CM derived from BM-MSCs stimulated with sEV-OCS or sEV-OCS-Cis. After 16 hours, the tube formation was examined using a light microscope, and images were analyzed using the ImageJ Angiogenesis Analyzer ver. 1.52 (National Institute of Health, MD, USA) [71]. The tube formation measured included junctions, nodes, meshes, and branches.

### 4.12. Ovarian Cancer Cells’ Migration

Low-invasive ovarian cancer cells Ovcar3 were cultured with CM derived from BM-MSCs stimulated with sEV-OCS or sEV-OCS-Cis. After 16 hours of stimulation, the Ovcar3 cells were tripsinized and cultured in a transwell migration assay (Corning Incorporated, Corning, NY, USA). Twenty-four hours after cell seeding, the Ovcar3 cells of the lower surface of the membrane (migrated cells) were fixed using methanol and stained with crystal violet (0.2%). Images were taken in a light microscope with a 10 × magnification, and the numbers of migrated cells were counted in five different fields per image.

### 4.13. Statistical Analyses

Statistical analyses were performed using the Kruskal–Wallis test followed by the Mann–Whitney test. Statistical significance was set at *p* < 0.05 or *p* < 0.01. The number of replicates was 3–6. The data were analyzed using Prism 6.0, GraphPad software (*GraphPad* Software, San Diego, CA).
